# Mobile social media use and life satisfaction among adolescents: a moderated mediation model

**DOI:** 10.3389/fpubh.2023.1117745

**Published:** 2023-11-29

**Authors:** Sujie Meng, Fanchang Kong, Wanghao Dong, Ying Zhang, Tingting Yu, Xiangdong Jin

**Affiliations:** ^1^Key Laboratory of Adolescent Cyberpsychology and Behavior, Ministry of Education, School of Psychology, Central China Normal University, Wuhan, China; ^2^School of Economics and Business Administration, Central China Normal University, Wuhan, China

**Keywords:** mobile social media use, life satisfaction, meaning in life, childhood psychological maltreatment, adolescents

## Abstract

**Introduction:**

Adolescence is a sensitive transitional period accompanied by great physical, mental, and behavioral changes. Therefore, maintaining physical and mental health is crucial to the growth and development of adolescents. As one of the important indicators of mental health, the influencing factors of life satisfaction have been widely concerned by scholars. In recent years, with the rapid development of Internet technology, mobile social media has penetrated into all aspects of adolescents’ life, which has a subtle impact on their physical and mental health. Existing studies have indicated that mobile social media use can affect adolescents’ life satisfaction. However, little is known about the mediating and moderating mechanisms linking this association. This study developed a moderated mediation model to examine the mediating role of meaning in life and the moderating role of childhood psychological maltreatment.

**Methods:**

A total of 1,198 adolescents across four provinces and municipalities of China completed questionnaires on mobile social media use, life satisfaction, meaning in life, and childhood psychological maltreatment.

**Results:**

After controlling for gender and age, the results demonstrated that mobile social media use was positively associated with life satisfaction and meaning in life among adolescents. Moreover, meaning in life fully mediated the association between mobile social media use and life satisfaction. Finally, the association between mobile social media use and life satisfaction, as well as that between mobile social media use and meaning in life, was moderated by childhood psychological maltreatment. Specifically, these associations are stronger for adolescents with high levels of psychological maltreatment.

**Discussion:**

These findings shed light on the important mechanism underlying mobile social media use’s effects on adolescents’ life satisfaction, which is helpful to formulate targeted measures for improving adolescents’ life satisfaction.

## 1 Introduction

With the development of mobile Internet technology, global users typically spend more than 4 h on mobile devices such as smartphones every day (1). Mobile devices have also become an important part and penetrated into all aspects of adolescents’ daily life. A public poll encompassing American teenagers showed that a majority of these participants are engaged in one or more social media activities by using smartphones (2). According to the newest national survey from China Internet Network Information Center (3), Chinese Internet users have reached 1,047 million by June 2022. About 99.6% of these users surf the Internet or message to others by mobile phones, and teenagers aged 10–19 years old account for 13.5% of Chinese Internet users. Mobile social media (MSM) not only refers to some traditional social media such as television but also applications with social functions specially developed for mobile devices, such as Microblogs and Wechat, reflecting the integrative function of mobility and socialization (4). MSM is indispensable for adolescents because it provides important ways to express themselves authentically, enhance communication, and reinforce intimate connections. Given that the use of MSM has become a behavioral habit among adolescents, as a typical behavior pattern in online life, MSM can have a significant impact on individuals’ development (5).

Adolescence is a transitional period in which physical, mental and behavioral factors are changing greatly (6). The attitude toward life among adolescents is an important mental health issue, drawing much attention in the past few decades. Life satisfaction refers to the individual’s subjective evaluation of their life (7), which has a significant relationship between the psychological and social functioning of adolescents (8). Many studies showed that higher levels of life satisfaction were associated with better quality of interpersonal relationships, more positive emotions, and higher self-esteem (8–10). Moreover, lower levels of life satisfaction can lead to more maladjustment, resulting in some externalizing and internalizing behaviors (11, 12). Adolescence is a critical period to form a sense of happiness and shape a positive attitude toward life, because it is accompanied by the profound biological, psychological and social developments (13). Accordingly, in order to promote and boost adaptive behaviors and healthy development among adolescents, it is crucial to identify influence factors and understand the underlying mechanisms of life satisfaction among adolescents. This research examines one potential mechanism between mobile social media use (MSMU) and life satisfaction, and if this association differs by one moderator.

### 1.1 Mobile social media use (MSMU) and life satisfaction

In the past decade, the issue of whether MSMU has a positive impact on mental health has received a great deal of attention. However, the results of relevant studies are contradictory: some researchers have found a positive correlation between digital technology use and mental health, whereas other studies have found that social media use impairs life satisfaction or that there is no link between the two variables [e.g., (14–17)].

Regarding the relationship between MSM and life satisfaction, social displacement theory suggests that online media hinders life satisfaction. Specifically, excessive social media use encrodes on people’s time for face-to-face social interaction in reality and reduces the utilization of social support in reality, which increases individuals’ sense of depression and alienation, resulting in negative consequences such as social anxiety and decreased life satisfaction (14, 18). While others hold different views (17, 19). From the perspective of the stimulation hypothesis, if adolescents spend more time with their friends through Internet communication, then their life satisfaction will be advanced due to the increased quality of friendship (17). Self-determination theory suggests that individuals have three basic psychological needs: autonomy needs, relatedness needs and competence needs (20). The autonomy needs refer to the individuals’ desire to make decisions or take actions autonomously, the relatedness needs involve the desire to connect with others, and the competence needs represent the need for the capability of mastering tasks and skills (20, 21). The satisfaction of basic psychological needs may facilitate better mental health, such as less loneliness, depression and anxiety (21). MSM provide individuals with a platform to express themselves autonomously, engage in social interactions, and connect with friends, which can help individuals meet these basic psychological needs (22). From this perspective, MSMU can positively impact adolescent life satisfaction primarily by fulfilling the psychological needs which are critical for well-being. Existing research has also verified the positive relationship between social media and life satisfaction due to more interactions with people and the improvement of online and offline social capital (5, 23). Likewise, a meta-analysis of 124 studies suggested that using social networking sites to interact with others, present oneself and entertain was a positive predictor for happiness and life satisfaction (24). Based on these existing results and the self-determination theory, this study hypothesized that MSMU is positively related to adolescent life satisfaction (Hypothesis 1).

As stated above, two questions are also worth discussing. The first concerns the underlying mechanism through which MSMU may enhance life satisfaction among adolescents. The second question is to explore whether the intensity or direction of this influence may vary with other factors. To address these gaps, the current research aims to examine the mediating role of meaning in life between MSMU and adolescent life satisfaction, as well as whether the mediating process may be moderated by childhood psychological maltreatment.

### 1.2 The mediating role of meaning in life

Meaning in life is considered as the perception of meaning, purpose and mission of life, and the degree to attempt to understand and realize the meaning (25). From the perspective of positive psychology, meaning in life is a critical psychological resource related to mental health, which is important to life satisfaction (26).

According to the self-determination theory, the basic psychological needs are powerful drivers of people’s mental health and behaviors (20, 27). When people are actively engaged on MSM, they may express themselves happily, increase social connection with others, or find common interests and support (17). Therefore, MSM has the potential to fulfill the needs for relatedness (27). Researchers found that social relationships are important sources of meaning in life (28). When individuals make more social connections, find more support and approval on social media, they may feel that relational needs are being met and feel an increased sense of meaning in their lives (29). In addition, it is worth noting the characteristics of the developmental period of adolescence, which is characterized by the establishment of self-identity and search for life goals (30, 31). MSM can provide platforms to explore and establish self-identity, and fulfill the need for autonomy (19, 27). With the gradual development of self-identity and the satisfaction of the need for autonomy, adolescents’ meaning in life can be effectively enhanced by the use of MSM (32). Furthermore, using MSM is a common style for adolescents to deal with daily events, accounting for a large part of their life (19). As a positive psychological resource, meaning in life can be influenced by daily life factors such as experiences and interpersonal relationship (33, 34). Although little research directly attended to the relationship between MSMU and meaning in life, researchers have found that Internet use is helpful for adolescents to enhance their relationship connections and intimacy, then increase quality of friendship, self-disclosure and sense of belonging, which are associated with greater meaning in life (19, 35, 36). Recently, Chen et al. (33) also reported the positive predictive effect of active social media use on meaning in life. Thus, we speculate that MSMU may facilitate the formation of meaning in life.

When adolescents feel that their lives are meaningful and search for missions of life actively, it is easier for them to build belief and hope, and such positive mental attitudes help enhance life satisfaction (30). A growing number of studies have examined the relationship between meaning in life and life satisfaction. For instance, a Chinese meta-analysis of 51 studies indicated that meaning in life is positively associated with subjective well-being, including life satisfaction (37). Empirical studies have also confirmed that meaning in life is a protective factor for life satisfaction (19, 38). Specifically, engaging in activities that could create meaning in life for adolescents is beneficial to a satisfying life. As stated above, it is possible that MSMU was associated with adolescents’ life satisfaction through meaning in life.

*Hypothes*is *2*: Meaning in life can mediate the relationship between MSMU and adolescents’ life satisfaction.

### 1.3 The moderating role of childhood psychological maltreatment

Using MSM may increase life satisfaction via meaning in life, but this effect may not exist in all adolescents equally. The ecological system theory suggests that the family as a micro-system plays a critical role in individuals’ development (39). Some studies found the moderating effect of family factors between adolescent behaviors and mental health, such as family cohesion, and parental attachment (40, 41). In our study, we focus on childhood psychological maltreatment, which is an important growth environment that cannot be ignored, and explore the potential influence of this variable on adolescents’ healthy development.

Childhood psychological maltreatment is defined as the continuous, inappropriate, and harmful parental behaviors by caregivers, involving indulging, spurning, neglect, and verbally threatening (42). Individuals mainly gain a sense of security and belonging from parental attachment, and those who have suffered psychological maltreatment by caregivers may perceive more family’s risks, and therefore their personal development is hindered (43). MSM provides more chances to express themselves comfortably, communicate with friends conveniently, and feel relaxed, making it easy to satisfy psychological needs and get social support (23). The theory of compensatory Internet use proposes that adolescents who are under stress may be more eager to alleviate negative emotions through the Internet (44), and moderate compensatory Internet use may contribute to maintaining pleasant moods and reduce negative moods such as loneliness (45). Due to the negative parental styles, individuals’ satisfaction with basic psychological needs is impeded or frustrated (46). Therefore, adolescents who experienced psychological maltreatment may feel a lack of basic psychological needs, and prefer to engage in social media activities in order to seek company or autonomy. In other words, the function of MSM may be particularly critical for those with maltreatment experiences. Previous studies mostly focused on maladaptive behaviors caused by compensatory network use, such as problematic mobile phone use (40, 47). However, from the perspective of motivation, adolescents want to seek support, obtain happiness and reduce negative emotions through the Internet, which is understandable and positive for their development (23, 45). A study has shown that teenage girls who are dissatisfied with the real friendship can search for friendship support on the Internet, and online friendship will play an active compensation role (48). Accordingly, we speculate that with the correct guidance, the Internet can indeed produce a positive impact on youngsters who have suffered from maltreatment by compensation. In the current study, we investigated whether the mediating process between MSMU and life satisfaction through meaning in life would be moderated by childhood psychological maltreatment.

*Hypothesis 3*: Childhood psychological maltreatment can moderate the mediating process between MSMU and adolescent life satisfaction. In particular, the association between MSMU and adolescent meaning in life and adolescent life satisfaction would be much stronger for adolescents with high levels of psychological maltreatment.

**Figure 1:**
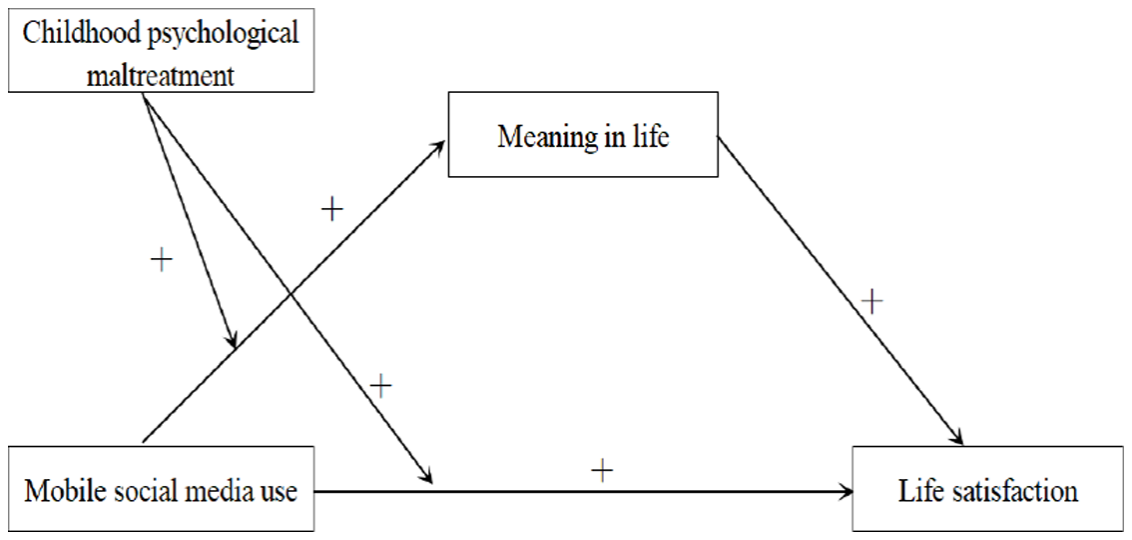
The proposed moderated mediation model.

The present study aimed to investigate the underlying mechanism between MSMU and life satisfaction. We proposed a moderated mediation analysis to examine (a) whether meaning in life would act as a mediator between MSMU and adolescent life satisfaction, and (b) whether this mediation process would be moderated by childhood psychological maltreatment. Specifically, greater mobile social media use would be associated with greater meaning in life and greater life satisfaction among highly maltreated adolescents than among less maltreated adolescents (see Figure 1).

## 2 Materials and methods

### 2.1 Participants and procedures

The participants were 1,250 students recruited from four middle schools in Anhui, Hubei, Jiangxi provinces, and Chongqing City, China. When responses were incomplete and regular (i.e., of same answer or certain regularity), it reflected this questionnaire was invalid. After exclusion, a total of 1,198 participants were valid. The mean age of the adolescents was 13.85 years (SD = 1.59), with males comprising 49% of the participants.

The present study was approved by the Ethics in Human Research Committee of the first author’s institution. After adolescent assent and teacher consent were obtained, participants filled out a series of questionnaires in the classroom, which was designed to collect data regarding some demographic variables and the research variables including MSMU, meaning in life, life satisfaction, and childhood psychological maltreatment. Well-trained graduate students described to participants how to complete the questionnaire using the standardized process. The researchers answered adolescents’ questions when needed. Participants were informed that all data were only used for research aims and this study was voluntary. No reward was presented to participants.

## 3 Measures

### 3.1 Mobile social media use

It was measured by the Adolescents’ Mobile Social Media Usage Behavior Scale, which was developed by Wang and Lei (4). It has been widely used in China and achieved good reliability and validity among Chinese adolescents (15). This scale comprised 15 items (e.g., “I post my life feeling on mobile social media”) and three dimensions, namely, interpersonal communication and presentation, information acquisition and sharing, and having fun and recreation. The scale was rated on a five-point Likert scale ranging from 1 (never) to 5 (at all times). Responses across the fifteen items were averaged, with higher scores indicating greater MSMU. The Cronbach’s alpha in this study was 0.91.

### 3.2 Life satisfaction

Adolescents’ life satisfaction was measured by the Satisfaction with Life Scale, which was developed by Diener et al. (49) and revised by Xiong and Xu (50). It included 5 items (e.g., “I am satisfied with my life”) and used a seven-point Likert scale ranging from 1 (strongly disagree) to 7 (strongly agree). Responses were averaged across all items, with higher scores representing higher levels of life satisfaction. This scale has demonstrated good reliability and validity among Chinese adolescents (51, 52). The Cronbach’s alpha in this study was 0.81.

### 3.3 Meaning in life

Adolescents’ meaning in life was measured by the Meaning in Life Questionnaire, which was developed by Steger et al. (25) and revised by Liu and Gan (53). This questionnaire consisted of 9 items and was divided into two dimensions--search for meaning (e.g., “I am looking for the purpose or mission of my life”) and presence of meaning (e.g., “I understand the meaning of my life”). Adolescents rated items on a five-point Likert scale ranging from 1 (strongly disagree) to 5 (strongly agree). Responses were averaged, with higher scores demonstrating greater meaning in life. The measure is widely used, and achieved good reliability and validity in Chinese adolescent samples (54). The Cronbach’s alpha in this study was 0.76.

### 3.4 Childhood psychological maltreatment

The childhood psychological maltreatment scale was used to assess how often their parents disciplined or neglected adolescents. The Chinese version of this scale was developed by Pan et al. (42) and widely used in China because it has achieved good reliability and validity among Chinese adolescents (51). The scale contains 23 items (e.g., “When I am sad or afraid, my parents do not comfort me”), with 5 dimensions, including terrorizing, neglect, spurning, interference and overindulgence. Adolescents rated items on a five-point Likert scale ranging from 1 (never) to 5 (always). Responses were averaged across the 23 items, with higher scores demonstrating higher levels of psychological maltreatment. According to a study by Liao et al. (55), scores ≥2 indicated adolescents have experienced psychological maltreatment in childhood, and a mean score of 1 demonstrated that they never experienced childhood psychological maltreatment. The Cronbach’s alpha for this present sample was 0.91.

### 3.5 Data analysis

First, in order to handle the missing data (less than 1%), mean imputation was applied (56). Then, descriptive statistics and correlation analysis were conducted to analyze the data. Third, this study used the PROCESS macro for SPSS (Model 4) to test the mediating role of meaning in life in the association between MSMU and life satisfaction (57). Next, the moderated mediation effect of childhood psychological maltreatment on the mediation process was tested by using the SPSS macro PROCESS (Model 8), which can indicate a direct effect and an indirect effect (57). The bootstrap confidence intervals (CIs) based on 5,000 random samples was used to examine the significance of the effects (57). If the 95% confidence intervals do not include zero, the effect is significant. Additionally, due to the potential relationship between adolescents’ gender, age, and mental health (58), we controlled for these demographic covariates during all analyses. Gender was dummy coded 0 for females and 1 for males. Finally, all continuous variables were standardized in the mediating and moderating analyses.

## 4 Results

### 4.1 Preliminary analyses

Because all the variables in this study were collected by questionnaire method, exploratory factor analysis was used to examine possible common method biases (59). Results showed that a total of 11 factors had eigenvalues over 1, and the first factor accounted for 17.97% of the total variance, and was less than 40%, which indicated that there were no significant common method biases in this study.

Then, descriptive statistics (means and standard deviations) and bivariate correlations across all study variables are presented in Table 1. As expected, MSMU was positively associated with adolescent life satisfaction (*r* = 0.09, *p* < 0.01). MSMU was also positively associated with meaning in life (*r* = 0.17, *p* < 0.001). Moreover, meaning in life was positively associated with life satisfaction (*r* = 0.26, *p* < 0.001). Finally, childhood psychological maltreatment was positively correlated with MSMU (*r* = 0.13, *p* < 0.001), and negatively correlated with meaning in life (*r* = −0.13, *p* < 0.001) and life satisfaction (*r* = −0.30, *p* < 0.001).

**Table 1:** Descriptive statistics and correlations.

Variables	*M*	SD	1	2	3	4
1.MSMU	2.79	0.87	–			
2.meaning in life	4.78	1.08	0.17^***^	–		
3.childhood psychological maltreatment	1.83	0.66	0.13^***^	−0.13^***^	–	
4.life satisfaction	4.03	1.40	0.09^**^	0.26^***^	−0.30^***^	–

*N* = 1,198. ^**^p < 0.01. ^***^p < 0.001.

We conducted the PROCESS macro (57) to examine the mediating effect of meaning in life on the relationship between MSMU and life satisfaction (Hypothesis 1).

As presented in Table 2, after controlling for gender and age, MSMU positively predicted life satisfaction (*b* = 0.09, *p* < 0.01) and positively predicted meaning in life (*b* = 0.17, *p* < 0.001). Hence, adolescents who used more social media reported higher levels of life satisfaction and meaning in life. Additionally, after controlling for gender and age, meaning in life was positively associated with life satisfaction (*b* = 0.23, *p* < 0.001). However, MSMU could not significantly predict life satisfaction (zero included in CI) after three variables were incorporated the regression equation. Therefore, meaning in life played a full mediation role in the association between MSMU and life satisfaction [indirect = 0.04, 95%CI = (0.02, 0.06)]. The mediating effect of meaning in life accounted for 48.39% of the total effect. This result partially supported Hypothesis 1.

**Table 2:** Mediation analysis.

Outcome variables	Independent variables	*b*	*SE*	*t*	*R^2^*	*F*
Life satisfaction	Gender	−0.08	0.06	−1.45	0.04	15.58^***^
	Age	−0.11	0.02	−6.17^***^		
	MSMU	0.09	0.03	3.16^**^		
Meaning in life	Gender	−0.07	0.06	−1.26	0.03	14.01^***^
	Age	−0.05	0.02	−2.77^**^		
	MSMU	0.17	0.03	5.96^***^		
Life satisfaction	Gender	−0.07	0.06	−1.19	0.09	29.68^***^
	Age	−0.10	0.02	−5.66^***^		
	MSMU	0.05	0.03	1.79		
	Meaning in life	0.23	0.03	8.32^***^		

*N* = 1,198. Bootstrap sample size = 5,000. ^**^p < 0.01. ^***^p < 0.001.

**Figure 2:**
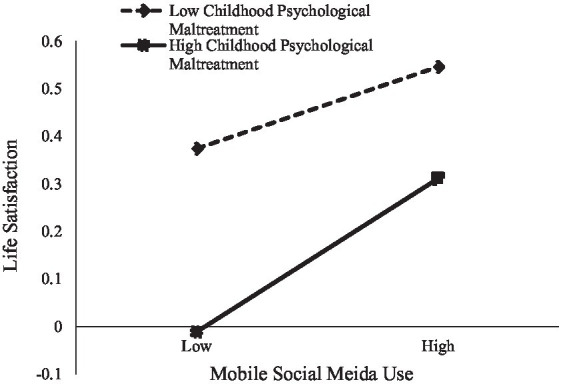
Childhood psychological maltreatment moderated the relationship between MSMU and meaning in life.

**Figure 3:**
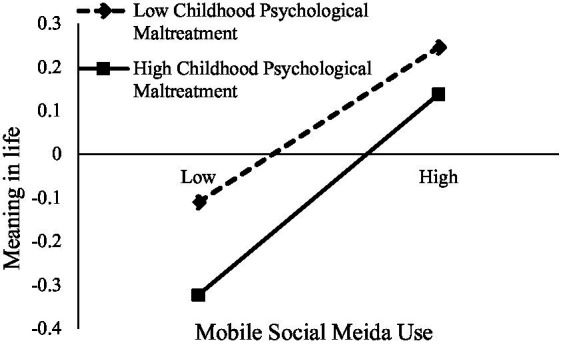
Childhood psychological maltreatment moderated the relationship between MSMU and life satisfaction.

We examined the moderating effects of childhood psychological maltreatment (Hypothesis 2). We used Model 8 of PROCESS macro (57) to conduct the moderated mediation analysis. Table 3 showed that after controlling for gender and age, the effect of MSMU on meaning in life was moderated by childhood psychological maltreatment (*b* = 0.05, *p* < 0.05). For clarity, we plotted MSMU on meaning in life, separately at low (1 *SD* below the mean) and high (1 *SD* above the mean) levels of childhood psychological maltreatment (see Figure 2). Simple slopes analysis (60) found that the effect between MSMU and meaning in life was stronger for adolescents with high levels of maltreatment (*b* = 0.24, *p* < 0.001) than adolescents with low levels of childhood psychological maltreatment (*b* = 0.13, *p* < 0.001). Finally, the direct effect of MSMU on life satisfaction was also moderated by childhood psychological maltreatment after controlling for gender and age (*b* = 0.07, *p* < 0.01). To be specific, simple slope tests (see Figure 3) indicated that for adolescents with higher levels of childhood psychological maltreatment, MSMU positively predicted life satisfaction (*b* = 0.17, *p* < 0.001). However, for adolescents with lower levels of maltreatment, this relationship was not significant (*b* = 0.03, *p* = 0.76).

**Table 3:** The moderated mediation analysis.

	*b*	*SE*	*t*	*R^2^*	*F*
*Meaning in life*				0.06	15.90^***^
Gender	−0.07	0.06	−1.32		
Age	−0.06	0.02	−3.43		
MSMU	0.19	0.03	6.68^***^		
Childhood psychological maltreatment	−0.17	0.03	−5.91^***^		
MSMU× Childhood psychological maltreatment	0.05	0.03	2.07^*^		
*Life satisfaction*				0.19	46.26^***^
Gender	−0.08	0.05	−1.55		
Age	−0.12	0.02	−7.42^***^		
MSMU	0.10	0.03	3.71^**^		
Childhood psychological maltreatment	−0.33	0.03	−11.97^***^		
MSMU× Childhood psychological maltreatment	0.07	0.02	3.15^**^		
Meaning in life	0.18	0.03	6.62^***^		

*N* = 1,198. Bootstrap sample size = 5,000. ^*^p < 0.01. ^**^p < 0.01. ^***^p < 0.001.

Moreover, we performed the bootstrap test to examine the conditional indirect effects of MSMU on life satisfaction through meaning in life. Results showed that for participants with higher childhood psychological maltreatment, MSMU had stronger positive effect on life satisfaction through meaning in life, and the effect size was 0.04 [*SE* = 0.01, 95% CI = (0.02, 0.07)]. In contrast, the indirect effect was weaker for participants with low levels of maltreatment, and the effect size was 0.02 [*SE* = 0.01, 95% CI = (0.01, 0.04); Table 4].

**Table 4:** Conditional direct effect of MSMU on life satisfaction at different levels of childhood psychological maltreatment.

Levels of childhood psychological maltreatment	Indirect effect	Boot *SE*	Boot LLCI	Boot ULCI
Conditional total effect of MSMU on life satisfaction				
Low (1 *SD* below mean)	0.03	0.04	−0.05	0.10
High (1 *SD* above mean)	0.17	0.04	0.11	0.24
Conditional direct effect of MSMU on meaning in life				
Low (1 *SD* below mean)	0.13	0.04	0.06	0.21
High (1 *SD* above mean)	0.24	0.04	0.17	0.31
Conditional indirect effect				
Low (1 *SD* below mean)	0.02	0.01	0.01	0.04
High (1 *SD* above mean)	0.04	0.01	0.02	0.07

*N* = 1,198. Confidence intervals that do not contain 0 are significant. SE, standard error, CI, confidence interval.

## 5 Discussion

The present study proposed a moderated mediation model to examine how MSMU is associated with adolescent life satisfaction and whether this association was varied by childhood psychological maltreatment. Results demonstrated that MSMU was indirectly related to life satisfaction via meaning in life after controlling gender and age. In addition, the first part of the indirect path and direct association were both moderated by childhood psychological maltreatment. Our findings extend our understanding of how and when MSMU is linked to adolescents’ life satisfaction.

### 5.1 The impact of MSMU on life satisfaction

The present study suggested that more MSMU predicted more adolescent life satisfaction, which was concordant with previous studies (5, 23). Due to the mobility and portability, using MSM has gradually become an important lifestyle among adolescents (19). Thus, adolescents get accustomed to using MSM to know the world and establish attitudes toward life. Our result revealed that when adolescents use MSM frequently, their development of life satisfaction could be advanced. This finding was also in accordance with the stimulation hypothesis and the self-determination theory, which proposes that adolescents can use online media to express themselves autonomously, engage in social interactions, and connect with friends, thereby meeting their autonomy and relatedness needs and increasing well-being (17). Therefore, using MSM can satisfy individuals’ basic psychological needs, then feel satisfied with life.

### 5.2 The mediating role of meaning in life

This present study revealed that MSMU was positively linked to meaning in life, which in turn was positively linked to adolescents’ life satisfaction. However, it is noteworthy that after introducing meaning in life into the regression equation, the direct effect of MSM on life satisfaction was missing. In other words, meaning in life is an important and complete mechanism linking MSMU and life satisfaction. This finding provides a perspective of how MSMU can lead to high levels of life satisfaction, that is, the reason is that they may maintain more meaning in life. To our knowledge, this finding is the first to point out that meaning in life can mediate the association between MSMU and life satisfaction in this area.

On the one hand, meaning in life can be influenced by daily relationships, events, and experiences (25, 28). Adolescents may use MSM frequently to obtain a greater quality of friendship or explore freely, and thus satisfy their psychological needs (19, 33, 35), which makes them more easily to search and build meaning in life. In this way, MSMU may promote and motivate adolescents to produce life meaning, which is consistent with prior studies (33). On the other hand, when adolescents possess more meaning or want to seek more meaning, they may build more positive attitudes towards life like hope, which is helpful to the improvement of life satisfaction (38). That is, the raise of life satisfaction can be driven by meaning in life when adolescents use MSM in their daily lives. This result supported the importance of meaning in life in accounting for individuals’ life satisfaction.

In a word, adolescents who use more MSM may have stronger missions and a sense of meaning and know what to do more clearly, thereby increasing a satisfied tendency and attitude towards life. This finding provides a basis for improving life satisfaction among adolescents. Moreover, it is beneficial for researchers who are focusing on the relationship between media use and adolescent mental health to use these theories and empirical evidence to propose research hypotheses.

### 5.3 The moderating role of childhood psychological maltreatment

Our results indicated that childhood psychological maltreatment significantly moderated the direct association and the first part of the indirect association between MSMU and life satisfaction. To be specific, the positive influence of MSMU on life satisfaction and meaning in life was stronger among adolescents who experienced more childhood psychological maltreatment. However, for those who have not experienced psychological maltreatment or have experienced less maltreatment, the relationship between these variables was weaker. When individuals experience harmful events, MSMU can evoke positive emotions, compensate those unsatisfied psychological needs and mitigate the negative impact on mental health (46), which validates the theory of compensatory Internet use, suggesting that negative events play an important role in impelling adolescents to engage in media use activities (44). On the contrary, in the case of the good parent–child relationship, it is common for individuals to get warmth and support from parents. Hence, when these adolescents use MSM, they no longer need mobile devices to satisfy their psychological needs, and then, one of the most important benefits of MSM may not apparent among adolescents who do not experience maltreatment. Accordingly, harmful childhood experiences are influenced by the use of MSM, and meanwhile, moderate the association between MSMU and mental health, that is, reinforce the positive effect of MSMU.

Moreover, our findings indicated that childhood psychological maltreatment, as a family environmental factor, can play a moderating role in the effect of other factors on individuals’ meaning in life. Meaning in life originated from the family (61). Those who use more MSM are more likely to keep high levels of meaning in life, and more maltreatment experiences can further strengthen the association between MSMU and meaning in life. One of the explanations may be that highly abused individuals experienced more neglect or assault in childhood, then showed less sense of meaning towards life, which in turn promoted them to maintain or seek meaning through online compensation. This finding highlights the functions of MSM to increase individuals’ meaning in life. Hence, when individuals are motivated to get positive feelings to keep enough inner resources by MSMU, this effect of MSMU may be profitable. No childhood psychological maltreatment experiences imply that families provide them with adequate support and inner resources. They obtain the meaning and missions in initial interactions and communications with parents, so they do not need to access additional resources and improve levels of life satisfaction through a non-offline pattern. To our knowledge, this is the first study demonstrating that this environmental moderator, can affect individuals’ development by influencing psychological resources. Our findings also explain the complex effects of MSMU on mental health (14, 18). MSMU probably does not act separately as a protective factor, but rather interacts with other factors like childhood psychological maltreatment to affect adolescent adaptive development.

### 5.4 Limitations and future directions

There are several limitations that need to be considered and discussed. First, this study collected data to measure variables through adolescents’ self-reports. Although results showed no serious common method bias, future studies should use multiple methods (such as interviews) and multiple informants (such as parents or peers report) to collect data more accurately.

Second, this study adopted a cross-sectional study design. Although valuable information has been found (57), we could not investigate any bidirectional or causal relationships. For example, adolescents who have high levels of life satisfaction and meaning in life may use MSM more frequently to enjoy life fully. The less satisfaction or meaning they possess, the less desire they have to engage in social media activities. Future studies should adopt a longitudinal or experimental design to further examine the association and possible mechanism between these variables.

Third, the current study examined only one mediating mechanism of MSMU on adolescents’ life satisfaction. Future research could consider other explanatory variables as mediators, such as psychological needs and self-esteem. Moreover, we also found one important moderator. Therefore, future research could explore the moderating effects of other family factors such as family functioning and attachment, and whether different environmental systems, such as peer relationships and teacher-student relationships would increase or reduce the influence which was brought about by MSMU.

Fourth, we used the aggregate score of the Adolescents’ Mobile Social Media Usage Behavior Scale to represent the level of MSMU. However, it is noteworthy that there are three dimensions of the Adolescents’ Mobile Social Media Usage Behavior Scale: interpersonal communication and presentation, information seeking and sharing, and having fun and recreation. This may reflect the fact that different characteristics of MSM may affect users differently through different mechanisms. Hence, it is essential to separate these three subscales and explore the differential effects across the three subscales in the future.

Finally, participants in this study came from four middle schools in China. In order to make our results more representative, future research should test whether these findings can be also applicable to other cultural backgrounds.

### 5.5 Implications

Despite these limitations, the findings of this study have important theoretical and practical implications. This study investigated the association between MSMU and adolescent life satisfaction from the perspective of the inner and external environment. Specifically, the study investigated the association between MSMU and adolescent life satisfaction and its underlying mechanism, and examined the moderating effect of childhood psychological maltreatment. These results are conduced to further deepen our understanding of the relationship and support the self-determination theory. Regarding the practical implications, firstly, our research underscores the vital role of MSMU in shaping adolescents’ life satisfaction. It is imperative for parents, educators, and administrators to acknowledge the profound impact that MSMU can have on the well-being of teenagers. To this end, it is crucial to implement necessary and effective measures aimed at guiding adolescents to use the Internet in a responsible and balanced manner. Such measures can help promote healthier and more satisfying lives for young individuals.

Secondly, our study highlights the mediating role of meaning in life in the relationship between MSMU and life satisfaction. To support the psychological well-being of adolescents, it is essential to educate them on how to identify meaningful content and experiences while using the Internet. This includes instilling values and guiding them to make deliberate and purposeful choices in their online activities. By doing so, we can empower adolescents to use the Internet as a tool for personal growth and healthy development.

Thirdly, we emphasize the significance of addressing childhood psychological maltreatment, which emerged as a key factor in our study. It is crucial to pay special attention to individuals who have experienced maltreatment during their formative years. For these adolescents, MSM can serve as a valuable means of seeking social support, maintaining a sense of meaning in life, and finding satisfaction. In light of our findings, it is vital to encourage these individuals to utilize MSM in constructive and therapeutic ways. This can help them mitigate the adverse effects of childhood maltreatment and foster positive psychological development.

Our research adds to the growing body of evidence that while overusing MSM can have detrimental effects on mental health, responsible and adaptive use can positively influence psychological development and counterbalance the negative impacts of family-related challenges. To this end, collaborative efforts between parents and schools are of paramount importance. By working together, they can help adolescents strike a balance in their Internet usage, mitigate the harm of childhood maltreatment, and instill positive and healthy attitudes, ensuring that young individuals navigate the digital landscape with wisdom and resilience.

## 6 Conclusion

In summary, the present study increases our comprehension of how and when MSMU is linked to life satisfaction. Results revealed that meaning in life was a full mediating mechanism through which MSMU was related to life satisfaction among adolescents. In addition, childhood psychological maltreatment could moderate the direct prediction of MSM on life satisfaction and the mediating effect of meaning in life. The moderated mediation model conducted in this study provides an integrated perspective and deepens our understanding of the mechanisms linking MSM and life satisfaction.

## Data availability statement

The raw data supporting the conclusions of this article will be made available by the authors, without undue reservation.

## Author contributions

SM collected and analyzed the data, wrote the original paper, and revised the manuscript. FK conceptualized and revisited paper. WD provided critical advice during the revision process and helped revise and rewrite the manuscript. YZ and TY provided constructive suggestions. XJ provided critical advice during the revision process and played a pivotal role in securing the necessary funding for this research. All authors contributed to the article and approved the submitted version.
